# What distinct opportunities could relational wellbeing as framework offer to advance wellbeing research? A purposive review of evidence

**DOI:** 10.3389/fpsyg.2026.1792773

**Published:** 2026-07-01

**Authors:** Angelique Wildschut, Hayley Abrahams, Yoliswa Ntsepe, Candice Groenewald, Sharlene Swartz

**Affiliations:** 1 Equitable Education and Economies Research Division, Human Sciences Research Council, Pretoria, South Africa; 2 Sociology Department, University of Pretoria, Pretoria, South Africa; 3 Faculty of Law, University of KwaZulu-Natal, Durban, South Africa; 4 Psychology Department, University of the Western Cape, Cape Town, South Africa; 5 School of Education, University of Cape Town, Cape Town, South Africa

**Keywords:** Global South, relational wellbeing, systematic review, wellbeing, young people

## Abstract

The notion of wellbeing emerged in resistance to deficit characterizations of people's lives in mental health theory and intervention. It is an approach that rejects compartmentalization and stigmatization, with the aim of centering and presenting a more holistic view of personhood. Informed by a new program of research on youth wellbeing in the Global South, this paper seeks to engage critically with the potential value of an emergent framework called relational wellbeing for advancing research on youth wellbeing. As a formative step, a scoping review was conducted and published in 2024 covering the period 2013–2022. To update and capture possible changes in the literature since then, the authors embarked on a mini review framed by the following two overarching questions: how has relational wellbeing been applied in the more recent academic literature, and to what extent is it being employed as a distinct approach? The mini review confirms that relational wellbeing, understood as a distinct framework, is emergent, but not solidified. The term continues to be used to challenge individualistic models of wellbeing, firmly positioning relationships, culture and context as central to wellbeing. However, the construct continues to lack unified operationalization—used variably as a theoretical lens, analytic framework and still quite strongly as a measurable construct. While this reveals a field conceptually rich and influential, it remains methodologically diffused. Insights across different sections of the review, however, provide guidance for prospective scholars hoping to expand research that applies a relational framework to foster greater understanding and positive impact on youth wellbeing.

## 1 Introduction

The study of wellbeing is a longstanding imperative in the social and health sciences, emerging in resistance to the deficit characterizations of people's lives in theory and intervention. Contrasting research that primarily studied poverty, dysfunction or exclusion, the theory of “wellness” emerged as a positive, aspirational and inclusive approach to understand lived realities and address complex needs (Bess and Doykos, 2014; Dolan and White, 2007; Gough and McGregor, 2007; Keyes, 1998, 2003; Mahali et al., 2018; White, 2010).

In positive psychology, wellbeing is often understood as consisting of two separate, but related facets, namely hedonic (i.e., “feeling good”) and eudaimonic (i.e., “functioning well”). Whereas, hedonic wellbeing relates to subjective wellbeing or life satisfaction (Diener et al., 1985, 2017), eudaimonic wellbeing is conceptualized differently by different theorists (Martela and Sheldon, 2019). Expanding the principles of hedonic and eudaimonic wellbeing, other conceptualizations and distinctions of wellbeing and its subsets have emerged including psychological wellbeing (e.g., positive personal functioning), social wellbeing (e.g., interpersonal, public and social functioning), and emotional wellbeing (e.g., positive affect and the ability to regulate emotions well) (Botha et al., 2019; Galderisi et al., 2015; Keyes, 2002, 2007). Relevant to discussion in this paper, Keyes (1998) further proposed five dimensions of social wellbeing, namely -actualization, -coherence, -acceptance, -contribution, and -integration that epitomizes the interpersonal, public and social functioning of one's life. This brief overview shows that wellbeing and the measurement of its constitutive parts are highly developed within this field, and relational wellbeing would be seen as one of the variables contributing to overall hedonic and eudaimonic aspects of wellbeing.

In the social sciences, slightly different terminology predominates, with a stronger focus on material- and/or subjective wellbeing. Material wellbeing tends to be seen as reflecting the assets people have, the state of physical welfare, standard of living, consumption, livelihoods, and wealth. Subjective wellbeing, on the other hand, is seen to entail people's perceptions and beliefs about what they have, along with their cultural values and the ideologies that influence their perceived ability to change circumstances. More recently, notions of multi-dimensional wellbeing have emerged, where wellbeing is seen as consisting of various domains (Johnson, 2009; Cooper et al., 2020) alongside the utilization of concepts of relational, social, and/or collective wellbeing.

## 2 Relational wellbeing

The construct of relational wellbeing has thus emerged from study across various disciplines including psychology, sociology, economics, sexology, social work, and even development practitioners, and this multidisciplinary application has yielded several definitions. Some scholars have defined relational wellbeing as the “quality of the relations between others” (Bess and Doykos, 2014; Yoo et al., 2021; Joshi et al., 2021), focusing on “peaceful co-existence” (Rillo-Albert et al., 2021), relational satisfaction or happiness (Lévesque et al., 2020; Leavitt et al., 2019). Others (McCubbin et al., 2012) take a more comprehensive view, stating that relational wellbeing encompasses “all dimensions of human ecology, including the family unit, ancestors, the physical and natural environment, extended family, adopted family, community, society, culture and the world” (McCubbin et al., 2012). Overall, though, the utilization of this construct, alongside others that tend to be used interchangeably in the social sciences, has become a critical counterpoint to notions of individual agency, highlighting the social, collective, and structural dimensions of wellbeing (Mahali et al., 2018; Roy et al., 2018).

Moving toward a more complex perspective on wellbeing, White and Jha (2023) posit that it emerges through the inter-relations of personal, societal and environmental structures and processes (White, 2010, 2017; White and Jha, 2020). In this regard, complimenting McCubbin et al. (2012) and others' conceptualization, seeing relational wellbeing as combining various dimensions of wellbeing into a unified theory to describe and address the interrelated factors that may compromise or facilitate wellbeing. As a framework it seeks to pave the way for an intentionally relational approach toward understanding, exploring and advancing wellbeing, arguing for a focus on the drivers of wellbeing (personal, societal and environmental), alongside a recognition of the co-constitutive nature of the following dimensions of wellbeing: having enough, feeling good, and being connected.

Taking these viewpoints together leads us to assert that applying White and Jha's (2020) conceptualization of relational wellbeing requires that, first, relational wellbeing must be engaged with as an ontology. Second, epistemologically, it should embrace a relational theory, relational thinking, and relational working. Third, it should embrace methodologies that facilitate co-creation, co-investment, and collaboration toward participatory and perhaps emancipatory outcomes (in research or intervention). Finally, through recognition and emphasis on societal structures, such an approach is likely to make policy more effective (White and Jha, 2023).

This conceptualization of relational wellbeing was adopted by a new program of research on youth wellbeing in the Global South^1^ in recognition that, while research into the wellbeing of young people has become a growing priority around the world, investigation often stops at describing their adversity, rather than making the effort to identify the strategies (both individual and collective) necessary to overcome their challenges (Swartz, 2021). A focus on Global South youth was further argued as important as, the majority (over 90%) of the world's young people live in the Global South (Ghai et al., 2022) and in comparison to their Northern counterparts, youth in the South generally experience increased population density; greater competition for opportunities; higher levels of income poverty, unemployment, and inequality; lower standards of living; and, often, higher rates of violence (Cooper et al., 2021). Furthermore, these empirical differences result not only in the ongoing exclusion from self-determination, wealth, and technology^2^ (UNDP, 2019) but also different capacities for responding to adversity and the scope of opportunities available to them.

The research programme thus seeks to contribute to this need for research into the wellbeing of young people, particularly those who live in the vulnerable regions of the Global South, drawing on the recent exciting insights and advances in scholarship on relational wellbeing. The argument is that relational wellbeing presents distinct opportunities for advancing social inquiry, understanding problems, and elevating the wellbeing of young people within contemporary societies faced by extreme economic, political, psychological, and ecological challenges disproportionate to other social groups.

Given the framework's emergent nature, as a formative step, a scoping review was conducted to better understand how the notion of relational wellbeing had been applied in the literature (see Wildschut et al., 2024). Through this formative review, which considered literature published between 2013 and 2022, we found firstly, that relational wellbeing was often used in reference to the quality of relationships; secondly, that it was commonly understood as a dimension of “multi-dimensional wellbeing”; and thirdly, that more recent applications appears to recognize relational wellbeing as a multi-faceted construct in and of itself (Wildschut et al., 2024). Relatedly, we found that while social- and collective wellbeing have been used interchangeably with relational wellbeing, research recognizes that the “collective,” “social,” and “relational” could be seen as completely distinct concepts. The review concludes that while the term relational wellbeing appears frequently in academic literature over the 10 years of evaluation, it is often in reference to the quality of relationships rather than as an encompassing framework or theory that positions relationality at the center of how wellbeing should be understood, engaged with, studied, promoted, and prioritized by, and in collaboration with, stakeholders and beneficiaries (Wildschut et al., 2024). The current paper then serves as an update to the previous review considering how relational wellbeing has been applied in recent academic literature.

## 3 Method

A systematic literature search was conducted following the PRISMA extension for scoping reviews (PRISMA-ScR) (Tricco et al., 2018). Scoping reviews are valuable in instances where the intention is to identify the types of available evidence in a given field and to clarify key concepts/definitions in the literature, to examine how research is conducted on a certain topic or field, identify key characteristics of factors related to a concept and finally, to analyze knowledge gaps and often is a precursor to a systematic review (Munn et al., 2018).

The search was conducted in Web of Science (WoS) using the search terms “relational wellbeing,” and the time parameters for the search were January 2021 to May 2025. WoS was identified as an aggregator database providing access to content from a range of publishers and disciplines and thus selected to allow us to explore a large scope of work, which might have been restricted in more focused databases.

**Figure 1:**
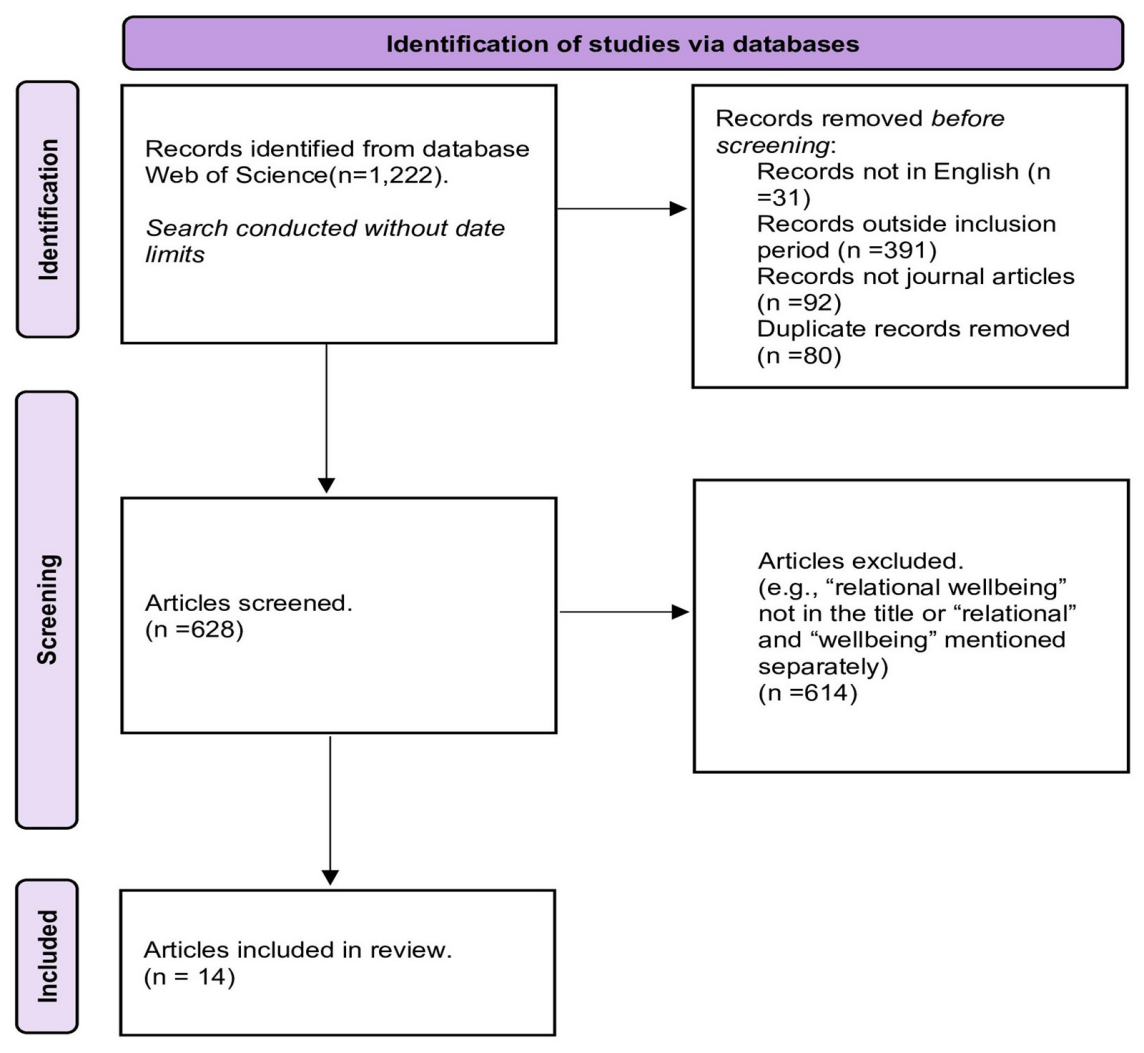
PRISMA diagram illustrating the identification, screening, and inclusion process leading to the final sample.

In addition to the timeframe, the inclusion criteria entailed peer reviewed articles, published in English and using the search terms of “relational wellbeing” consecutively (as a Boolean phrase), and not as standalone subjects, in the title and abstracts. The latter was a purposive criterion as we were explicitly interested in assessing how the notion of “relational wellbeing” as a construct has been applied. Although context is important, we did not apply any further parameters in terms of location, as we wanted to align with the methodology applied in the previous review, but also as the geographic location of the application is an area of analysis and investigation we would like to pursue as the program of research develops. As illustrated in Figure 1 the literature search resulted in a total of 1,222 articles being retrieved from the database. In the identification phase, a total of 594 papers were excluded because they were; (1) not published in English (*n* = 31), (2) outside of the inclusion period (*n* = 391), (3) not peer reviewed (*n* = 92), or (4) duplicate records (*n* = 80) (refer to Figure 1).

Further, the abstracts and titles of the remaining articles (*n* = 628) were screened, paying particular attention to whether the term “relational wellbeing” (as Boolean phrase) was utilized, or whether the concepts of “relational” and “wellbeing” appeared separately. A total of 614 records were excluded at this point. This resulted in a total of 14 eligible papers that met the inclusion criteria, which were subjected to full-text analysis to examine how relational wellbeing was conceptualized and applied. The analysis considered (1) how relational wellbeing was utilized, (2) key theoretical foundations of its use, (3) key findings, and (4) research gaps identified.

## 4 Results

**Figure 2:**
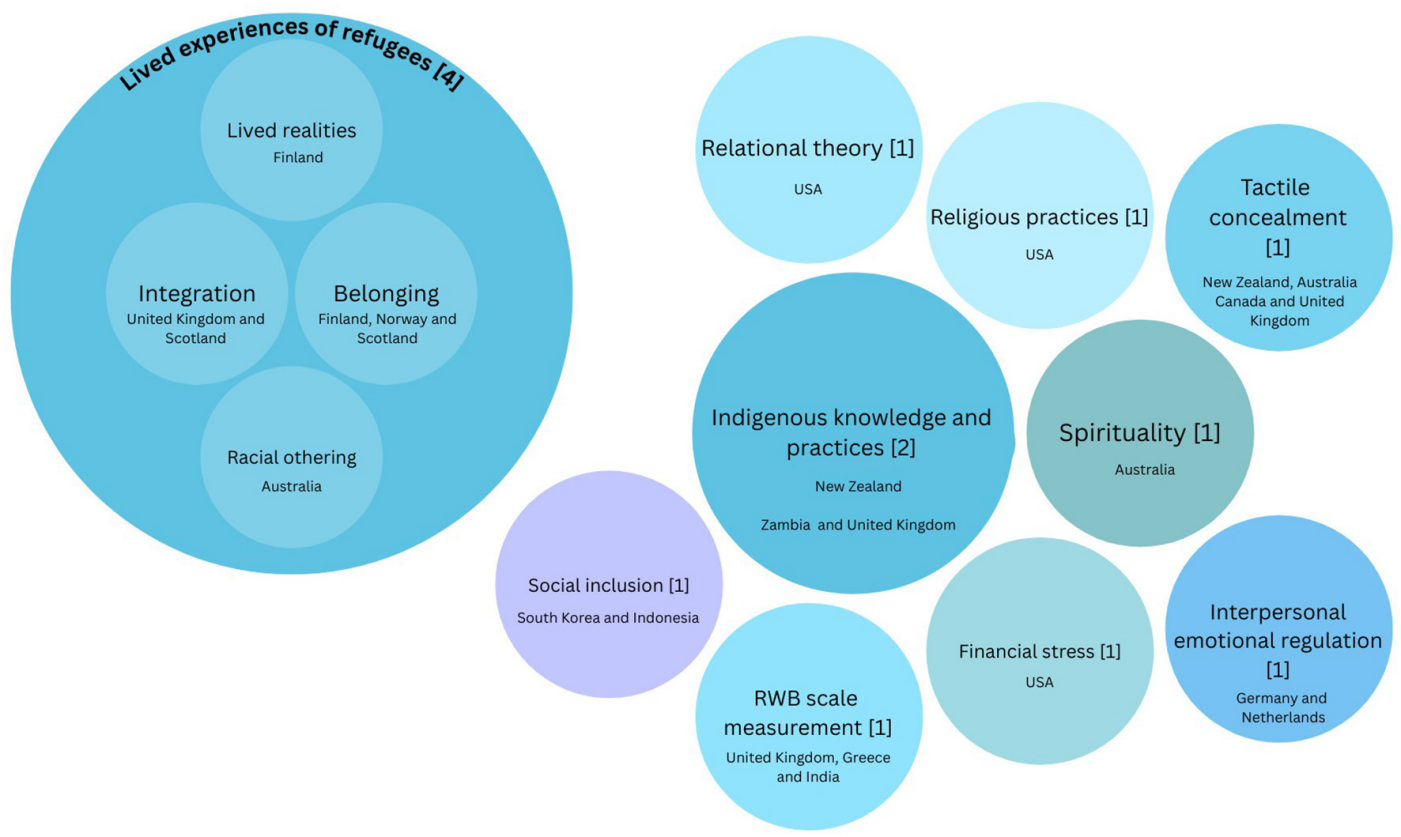
High-level themes covered in the sample.

Our analysis shows coverage traverses a wide range of journals spread across disciplines of psychology, social sciences, religious, organizational, family and social indicator studies (see Supplementary Appendix A for sample list and Supplementary Appendix B for the data extraction table). Consequently, the concept of relational wellbeing was employed to study various topics, as illustrated in Figure 2. Interestingly, four studies explored relational wellbeing as experienced by young refugees in different contexts, two studies focused on indigenous knowledge and practices, and further two studies considered relational wellbeing and its association with spirituality and religious practices, respectively. The focus areas of the remaining six articles were diverse and are presented below. The next sections of the paper will provide further detail, engaging more robustly with how the construct was applied and insights emerging from the detailed analysis.

### 4.1 Relational wellbeing consistently centers the quality of relationships

Corroborating our previous review, we found that relational wellbeing primarily continues to be conceptualized as the quality and health of interpersonal relationships, particularly within family, romantic, and social contexts. However, whereas the previous sample (2013–2022) displayed a specific focus on (1) the state of relationships existing between men and women in heterosexual relationships, (2) wider familial relationships, between siblings and parent-child and/or sibling-child relationships and (3) relational wellbeing in the form of collegial relationships in the workplace, in this sample, the context of community and communal relationships appears more prevalent.

For example, Dollahite et al. (2022) frame relational wellbeing in terms of the quality and nature of family ties, showing that increases in home-based religious practices correlate with greater familial cohesion and improved relational satisfaction. This perspective situates relational wellbeing as an outcome of shared relational processes, with family interactions serving as both the site, and measure, of wellbeing. Similarly, Kelley et al. (2023) conceptualized relational wellbeing as a measurable outcome to understand how family and romantic relationships are affected by changes in financial stress. In this view, relational wellbeing is seen as mediating the relationship between economic stressors and psychological outcomes, further reinforcing the idea that relational wellbeing can be assessed through observable relationship quality.

Pauw et al. (2024), examining relational wellbeing as an outcome, assessed it within the context of romantic relationships as well, by measuring explicit short-term outcomes related to connection and satisfaction. The focus is on how individuals regulate their emotions with the help of others, a phenomenon referred to as interpersonal emotion regulation (IER). The study investigates the effectiveness of various IER strategies for improving emotional and relational wellbeing in daily life among romantic couples. This approach powerfully illustrates how moment-to-moment interactions, rather than aggregate assessments of relationship quality, actively shape wellbeing.

Jones et al. (2023) on the other hand, utilizes the construct of relational wellbeing to explore the relationship between concealment and wellbeing within the context of professional relationships, considering feelings of acceptance, integration, and contribution. Drawing from Scott's “hidden transcripts” and Keyes's five dimensions of social wellbeing, the study reveals a complex and often contradictory relationship between tactical concealments and relational wellbeing, presenting both generative and destructive pathways. Here, relational wellbeing is defined as a socially constructed appraisal of one's functioning in society, which can be both enhanced and complicated by the tactical use of concealment. They established that academics employ tactical concealment strategies to preserve supportive working relationships, like coffee machine chats, which enhance relational wellbeing by providing needed social acceptance, social integration, and social contribution.

Increasingly, however, in the sample of papers reviewed, the community as context for relational wellbeing is being amplified. Here for example Nicholson et al. (2025) explores relational wellbeing within the context of Māori community support workers, likening it to the concept of *Hauora* as underpinned by principles centered on social units that provide reciprocal spiritual and emotional support. As such, the quality of relationships is assessed by whether interactions reflect the ethical force of life, called *hau*, or the disconnection which diminishes life energies, rendering people emotionally vulnerable and sick. Caring and being cared for is considered a critical reciprocal activity for relational wellbeing. A powerful contribution here is in illustrating that indigenous knowledge systems are operationalizing relational wellbeing in ways structurally distinct from dominant individualistic Western models. White and Jha's (2020) contribution similarly highlight the importance of community, in their empirical work on kinship networks in Zambia, where relationships between birth and foster parents, were intertwined, existing within a context of tensions and trade-offs experienced by individuals, within a collective ideology that emphasizes solidarity. While the collective ideology in this context emphasizes collective agreement in placing children in foster homes, the children may experience emotional abandonment, despite the material benefits of the placement.

Kohli et al. (2023) also explore relational wellbeing within the context of community in their focus on the integration of young refugees. In finding that young people's integration experiences are layered and mutual, with strong peer, familial, and community ties serving as a foundation for both emotional security and broader psychosocial adjustment, their work emphasizes mutuality, emotional connection and community ties as central to integration. They approach relational wellbeing as a multidimensional construct, conceptualizing it as one layer within broader social and psychological wellbeing. Their findings indicate that social connectedness and mutual recognition are crucial determinants of young people's overall wellbeing, reinforcing the idea that relational wellbeing interacts dynamically with other wellbeing domains.

Gaines et al. (2024) is concerned with the measurement of relational wellbeing, with their overall approach aligning with much of the papers in this category in viewing or investigating relational wellbeing as an outcome variable and conceptualizing it as a dimension of wellbeing. They explore the construct and criterion-related validity of an eight-item relational wellbeing scale across diverse cultural contexts, confirming socio-cultural context as a significant predictor of relational wellbeing. Another quantitative comparative paper by Seda et al. (2023) intends to fill the gap in examining how vertical (assets, income level, occupational background and educational level) and horizontal (gender group, religious affiliation, region of origin, race/ethnicity/nationality) forms of social inclusion have impacted on wellbeing in its relational forms; specifically, the capacity to trust others, the degree of interaction and pro-activity in communal participation. Here, relational wellbeing is defined as an individual's sense of social happiness, which is cultivated through subjective perceptions of trust, personal interactions with fellow individuals, and social engagement in communal activities. These two papers align on their suggestion that relational wellbeing should be viewed as a unified factor, rather than consisting of two distinct components (Gaines et al., 2024; Seda et al., 2023).

### 4.2 Relational wellbeing as embodied, spiritual, and place-based

The previous review confirmed that relational wellbeing is most commonly understood in relation to discussion of multi-dimensional wellbeing, which recognizes wellbeing as constituted by different dimensions, of which relational wellbeing is one. While the later papers in the previous review did show growing sophistication in recognizing relational wellbeing as a multi-facetted construct varying based on culture and other aspects, in this sample, the concept of relational wellbeing was much more clearly articulated as associated with layered, contextual, and spiritual practices, which did not emerge strongly from the previous review (three papers).

Haswell (2023) is an example of how the centrality of nature as a site for fostering wellbeing is being recognized through exploring the relationship between nature contact, wellbeing and belonging in the resettlement experiences of young refuges in Finland. Employing the relational wellbeing approach of White and Jha (2021) the paper considers how subjective, material and relational dimensions of wellbeing arise and interrelate within refugees' encounters with nature and how these link to their development of a sense of belonging. A critical insight here is that people's sense of belonging emerges from the holistic, simultaneously experienced, physical, affective, cognitive and social dimensions of encountering people, objects and places. The strength of people's sense of belonging is seen as depending on the extent to which those dimensions generate circumstances of feeling good, having enough and being connected and a strong recognition of nature as a site where encounters provide healing, continuity and hope across time. Hauw and Halafoff (2025) also touches on nature and community while foregrounding the embodied and spiritual elements of relational wellbeing. Drawing on theories of lived religion and everyday spirituality, they show how relational wellbeing, as a holistic, embodied and ethically driven concept explicates how dance facilitates deep connections within individuals, between individuals in community and with the nature and sacred world, highlighting spirituality as deeply social, ecological, and affective.

The embodied character of wellbeing also emerges quite strongly in Katisi et al.'s (2024) exploration, where the authors employ physical objects to access respondent's experience and conceptualization of relational wellbeing. Utilizing a participatory methodology, respondents were asked to bring objects that show how they experience and choose to express relational wellbeing, conceptualized as constituted by three aspects, feeling good, being connected, and having enough. The findings revealed three themes of experience and expression, highlighting the overlap between old and new social ties, time and space across these three aspects. The importance of developing complex research and intervention methods able to capture relational wellbeing, or “tapestries of young refugee experiences” is powerfully illustrated in this research paper. While highlighting different elements, collectively these papers speak to a greater acknowledgment in recent work of the layered, place-based and embodied nature of wellbeing.

### 4.3 Relational wellbeing not always positive—Can reproduce power, exclusion, and marginalization

While clearly acknowledged as a more positive and holistic orientation to the notion of health and wellbeing in our previous sample, our mini review update highlights finally an increasing acknowledgment of the potentially negative implications of relational wellbeing. Here, for example, Jones et al.'s (2023) exploration shows how increased relational wellbeing within the workplace can enhance integration with colleagues but can also lead to social disintegration in relation to family ties, if time spent at work is excessive. Nicholson et al. (2025) is also relevant here as they explore, within the context of Māori community support workers, the quality of relationship with clients, finding these to be defined in familial terms, showing an overlap between professional and familial relationship ethics. For the Māori community support workers, a challenge arises when relationship quality is undermined by imposing rigid Western models of care and professional boundaries that prohibit the expression of tikanga Māori (Māori customary practices), viewing close bonds as unprofessional.

Molla's (2023) contribution is also noteworthy in its consideration of racial othering and its effect on wellbeing. Two aspects of relational wellbeing are investigated: the capacity to move in public without fear or shame and the ability to feel a sense of belonging to the place where one lives in. Again however, relational wellbeing is conceptualized as an outcome variable as illustrated in the key research question; “how has the racialization of violence affected the opportunity for African refugee youth to achieve relational wellbeing?.” The paper concludes that young people have diminished wellbeing opportunities when they lack equal worth and respect and face difficulties in establishing meaningful relationships with others—when they are not free from alienation, deprivation and domination. Hauw and Halafoff's (2025) contribution also recognizes an insufficient focus on issues of safety, inclusion and the power dynamics and exclusions inherent in relational wellbeing within current research within spiritual communities.

## 5 Discussion and conclusion

As detailed above, where relational wellbeing is employed as framework these applications appear to surface: (1) how belonging is built through layered place-based and affective relationships (Kohli et al., 2023), (2) the importance of nature as a site for wellbeing (Haswell, 2023), (3) the ability of non-verbal methods to offer deep access to lived experiences (Kohli et al., 2023; Katisi et al., 2024) and (4) wellbeing as a process and not a state, unfolding relationally over time through changing contexts and interactions (White and Jha, 2023). It is noteworthy that many of the more recent papers in the sample emerge from a Special Issue on relational wellbeing for young refugees, edited by White and Jha. These were thus explicitly challenged to engage with relational wellbeing as framework. However, the application of this approach within the context of youth experience as they seek belonging and integration, appears to allow for access, deep reflection, and narration of personhood. Here, the recognition of the co-constitutive nature of wellbeing emerged strongly, unencumbered by the need to compartmentalize domains of wellbeing according to pre-defined areas. This appeared to allow a presentation of wellbeing that illustrates complexities in experience where there are overlaps in time and space, old and new social ties and between the three dimensions of wellbeing; having enough, feeling good and being connected. Within increasingly shifting and complex contexts that characterize the realities of young people, such an approach can offer powerful opportunities to access and illustrate the complexity of youth wellbeing. Such approaches become important in recognition of the complex navigations of young people as they seek livelihoods, contend with assaults on their mental health, experience the impact of climate change and the confluence of much of these factors with increasing digitalization.

At a more general and collective level, it is clear from the mini-review that relational wellbeing continues to be applied variably, as a theoretical lens, analytic tool, and measurable variable. Some employ it conceptually (Jones et al., 2023; Wesner et al., 2025); others use it analytically in qualitative research (Dollahite et al., 2022; Kelley et al., 2023); some continue to operationalize and measure it quantitatively (Gaines et al., 2024; Seda et al., 2023), with diverging views on whether it should be measured as a unified (Gaines et al., 2024) or multi-facetted indicator (Jones et al., 2023; Seda et al., 2023). Where relational wellbeing is dealt with as an outcome or mediating variable, this aligns with conceptions of relational wellbeing as a facet of overall wellbeing, and as noted, few employ relational wellbeing as framework (White and Jha, 2020; Kohli et al., 2023; Haswell, 2023; Katisi et al., 2024). This reveals relational wellbeing as conceptually rich and influential but methodologically diffuse, resulting in a lack of standardized definitions or accepted measures, broad interpretive flexibility and opportunities for refining conceptual and methodological clarity in its application. Relational wellbeing is therefore widely valued but inconsistently applied, suggesting a field in an emergent stage of defining what relational wellbeing is and how it can (or should) be operationalised.

However, across nearly all the papers in this review, relational wellbeing is consistently used as a counterpoint to individualistic approaches or understanding of wellbeing by positioning relationships, culture and context as central to wellbeing. Authors emphasize that wellbeing emerges between people, within families, communities, cultural systems, spiritual traditions, and sometimes within the relationship between nature and human interaction. This solidifies relational wellbeing as a relational, contextual, and often holistic construct used to foreground the significance of (1) indigenous and multiple cultural worldviews, (2) emotional connection, belonging and mutuality, (3) relational practices, explicated in forms such as caregiving, ritualists or communication, and (4) ecological, collective and systemic influences. This has strengthened a shared shift across a range of disciplinary literatures toward understanding wellbeing as embedded, interdependent, and socially shaped, rather than only an internal psychological state. The advantages need to be carefully brought into conversation with its drawbacks, which could relate to the lack of comparability, standardization and time and resources required for implementing such a complex approach. We are confident that a broader scope and depth of empirical application of relational wellbeing as framework will assist in crystallizing its potential value for wellbeing research.

## Footnotes

1 This research programme is called u'Good!? and it aims to advance youth wellbeing in the Global South using a relational approach. The program supports projects that encourage researchers to adopt and test a relational approach to wellbeing, and purposively focuses on the intersections and inflection points in young people's lives, such as livelihoods, mental health, digitalization, and climate change. The program is multi-year and multi-country, funding 23 research projects across nine Global South countries.

2 Being on the technological periphery has vast implications for young people in the Global South, especially since access to information drives employment and wealth creation.
