# Supplementary material for: What distinct opportunities could relational wellbeing as framework offer to advance wellbeing research? A purposive review of evidence
Source: Front Psychol. 2026 Jul 1;17:1792773. doi: 10.3389/fpsyg.2026.1792773 (PMC13368665; doi:10.3389/fpsyg.2026.1792773)
Supplement: Supplementary file 1 [file Data_Sheet_1.pdf]

Citation (Author, Year, Title, Source)	Abstract	Use of Relational Wellbeing	Key Theoretical Foundations	Study Population (Age, Context)	Geographical Context	Methods Used	Key Findings	Identified Research Gaps
Hauw, S. & Halafoff, A., 2025. **5Rhythms dance as lived spirituality: women, embodiment, and relational wellbeing**. *Journal of Contemporary Religion*.	This study examines women's experiences of 5 Rhythms dance in Melbourne, Australia, as a lived, embodied, and relational spiritual practice. Drawing on theories of lived religion and everyday spirituality, it explores how the practice promotes personal	RWB in this paper is a holistic, embodied, and ethically-driven concept that underpins how 5Rhythms dance facilitates deep connections within individuals, between individuals in community, and with the natural and sacred world.	Lived religion (McGuire, 2008); everyday spirituality (Ammerman, 2013a, 2013b); bodily becoming (LaMothe, 2012); ethical spirituality; critique of neoliberalism and patriarchy.	Adult women (range not specified), urban context.	Melbourne, Australia.	Qualitative narrative methodology (interviews and participant observations), thematic analysis.	5Rhythms promotes personal and relational wellbeing through embodied spiritual practice. Spirituality is deeply social, ecological, and affective.	The authors identified that safety and inclusion in spiritual communities are not critically examined, leaving power dynamics and exclusions within relational wellbeing insufficiently addressed.

	healing, ethical relationality, and collective wellbeing.							
Kelley, H.H., Lee, Y., LeBaron-Black, A., Dollahite, D.C., James, S., Marks, L.D. & Hall, T., 2023. **Change in financial stress and relational wellbeing during COVID-19: exacerbating and alleviating influences.** *Journal of Family and Economic Issues*, 44(1), pp.34–52.	Guided by the FAAR model and using a panel survey of 1510 US adults during Covid-19, this study explores how changes in financial stress influence relational wellbeing, showing complex effects on conflict, closeness,	In this paper, RWB is being conceptualized and used as a practical measurable outcome to understand how family and couple relationships are affected by changes in financial stress, particularly during the Covid-19 pandemic. The paper emphasizes that RWB can improve or deteriorate	Family Adjustment and Adaptation Response (FAAR) model (Patterson, 1988); Concepts of maladaptation and bonadaptation; Prior frameworks on COVID-19 and family relationships (e.g., Prime et al., 2020).	1510 adults, wide age range (18 - over 70), 71% married, 70% parents. Broad rep across income, education, race/ethnicity, and religious background.	America (participants from 40 states).	Mixed methods: Quantitative survey and qualitative open-ended questions.	The study shows that financial stress has both harmful and potentially constructive effects on relational wellbeing. While increased stress often led to more conflict, it could also bring families closer if buffered by strong financial,	**1.** The authors notes that there is limited research on COVID-19's impact on RWB. **2.** A scarcity of mixed-methods studies limits understanding of the complex, nuanced ways financial stress impacts RWB. Most prior studies rely solely on

	and happiness.	depending on resources, meaning-making, and coping strategies, thus providing a nuanced understanding of wellbeing in relational contexts.					relational, or emotional resources.	quantitative data.
Dollahite, D. C. *et al*. (2022) **'Changes in Home-Centered Religious Practices and Relational Wellbeing following the Initial Onset of the COVID-19 Pandemic'**, *Marriage &*	This study explored how changes home-centred religious practises during early Covid-19 closures influenced family relational wellbeing in the US. It examined	RWB is conceptualized as the quality and nature of family relationships. The authors use and measure relational wellbeing through three primary indicators: Family relational	Prime et al.'s (2020) Covid-family wellbeing model; Generative Devotion Framework (Dollahite and Marks); family stress theory; religious coping literature (Pargament et al.): post-traumatic growth.	1510 US adults, 50% female; diverse religious affiliations; ages 18 - 70+.	America (participants from 40 states).	Mixed methods: Quantitative survey and qualitative open-ended questions. Cross-sectional design.	Increasing regular home-based religious practices was associated with greater family closeness and positive perceived lasting effects. Family	**1**. The study used retrospective self-reports due to the unexpected nature of the COVID-19 pandemic. There is a lack of true longitudinal studies examining how relational wellbeing

Family Review, 59(2), pp. 65–94.	emotional closeness, relational conflict, and perceived lasting family impact, using survey data from 1510 participants.	conflict, emotional closesness, and perceived long-term impact of the COVID-19 pandemic on relationships.					prayer, scripture study, and home worship fostered resilience and relational wellbeing during Covid-19.	evolves before, during, and after major social disruptions like the pandemic. **2.** The study primarily surveyed adults. There is a gap in understanding children's and adolescents' perspectives on family relational wellbeing and spiritual practices during crises like COVID-19.

Wesner, C.A., Around Him, D., Ullrich, J.S., Martin, L., Denmark, N., Russette, H., Lee, K., Sarche, M., Asdigian, N.L., Barnes-Najor, J. & Whitesell, N.R., 2025. **Co-creating a conceptual model of Indigenous relational wellbeing in early childhood: Planting seeds of connectedness.** *Infant Mental Health Journal*, 46(2), pp.115–132.	This article shares the co-creation of a conceptual model of indigenous relational wellbeing, reflecting American Indian and Alaska Native worldviews. Guided by Indigenous research methodologies and a Community of Learning (CoL), the model applies an Indigenous connectedness framework to early	This paper conceptualizes RWB as a culturally embedded, holistic, and lifelong process of connectedness shaped by community, spirituality, and ancestral knowledge.	Indigenous connectedness framework (Ullrich, 2019); Indigenous research methodologies; Two-eyed seeing; Braided river (He Awa Whiria); Critical theory; Community of Learning.	Not empirical.	America.	Conceptual and participatory, literature reviews, collaborative development, group concept mapping.	**1.** Western measures of RWB are culturally misaligned. **2.** A conceptual model of indigenous ERW was developed: authors co-created a visula model that: Highlights four domains of connectedness: family, intergenerational, community, and environment; hows how relational wellbeing unfolds	**1.** Authors have identified a conceptual gap: there is a lack of culturally grounded RWB models. **2.** There is also a need for broader definitons of RWB: Research has overly emphasized the parent-child dyad, neglecting extended family, community, and environmental relationships essential to Indigenous

	childhood development. It emphasizes spirituality, culture, and ceremony as foundational to wellbeing, and the intergenerational, community, and ecological contexts of child development.						across the lifespan; Centers ceremony, culture, and spirituality as foundational.	conceptions of wellbeing.

Kohli, R.K.S., Sullivan, P. & Baughan, K., 2023. **Drawing together in Scotland: The opportunities and challenges for young refugees within a 'relational wellbeing' approach to integration**. *Social Sciences*, 12(12), p.666	This article explores how young refugees in Scotland experience integration through a relational wellbeing lens. Based on the Drawing Together project, it highlights integration as both a process and an outcome rooted in hospitality, reciprocity, and multiple belongings.	The paper uses RWB as a lens to explore how young refugees experience integration in Scotland. RWB in this paper is a holistic, experiential, and dynamic framework for understanding integration—a lived, relational, and often fragile journey shaped by mutuality, emotional connection, and community	RWB (White, 2008; White and Jha, 2020, 2023); Refugee integration frameworks (Ager and Strang, 2004, 2008); Acculturation theory (Berry, 1997); Hospitality and reciprocity.	53 young refuges, aged 18 to 30 years, 26 men and 25 women. Mostly students or in low-paid work.	Scotland, UK.	Mixed methods: Longitudinal qualitative study; interviews; ecomaps; art workshops.	Integration is layered and mutual. Social ties, faith education, employment, and art-based expression contribute to resilience and wellbeing.	**1.** The authors uses White & Jha's RWB framework (having enough, being connected, feeling good) to refugee integration but also expose how RWB is underutilized in migration research. **2.** Intersections between environmental belonging and RWB are overlooked.

		embeddedness						
White, S.C. & Jha, S., 2023. **Exploring the relational in relational wellbeing.** *Social Sciences*, 12(11), p.600.	RWB is conceptualized as an **integrative approach** that places the relational, rather than the individual, at the centre of analysis. It views being in wellbeing as a process that unfolds over time,	Conceptualizing persons as **relational subjects,** meaning individuals are subjects of their own lives, forged in their relationships with others and their material and social contexts, rather than independent entities. Relationships are not just	RWB primarily rooted in **relational social science and a relational ontology** stating that persons are relational subjects. The approach embraces **multiple dimensions** of human experience, where a unique individual identity coexists with being intertwined with others, and	Men and women of Chiawa village. Women participants in UK based workshops	Chiawa village in Zambia and NHS Hospitals in the UK	Mixed methods - surveys, case studies and workshop data	1. Persons are Relational Subjects, not independent individuals. 2. That wellbeing is best seen as a process that emerges over time through interactions between people, other	The paper suggests that the RWB approach needs to be tested and extended in different contexts. 2. The authors also invite others to explore RWB using other methods.

	emerging from interactions between people, other beings, places, and things, rather than an internal state or an aspect belonging solely to an individual. This process-oriented view suggests that wellbeing may produce durable equilibrium states that are flexible manifestati	constitutive of people but also serve as conduits for meeting diverse needs, extending beyond mere psychological or emotional support to material concerns and terms of interaction.	expressions of self-shift with context. Drawing on theorists like Foucault, and feminist and anti-racist work, the approach recognizes that **power** is exercised through innumerable points within relationships, and that social differences like gender or race are not just given but "done" and "achieved" through everyday social interactions.				beings, places, and things, rather than a segmented internal state or a distinct aspect of an individual's life. 3.The concepts and methods used in research profoundly shape the representations of persons and wellbeing. 4. Wellbeing experiences are found to be the	

	ons of underlying processes.						outcome of the constant interaction of underlying drivers: personal, societal, and environmental. 5. That relationships are not neutral but serve as conduits of power and are integral to the making of identities. 6. Despite its theoretical anchoring in research from the Global South, the	

							source finds that the RWB approach is transferable to Global North contexts, as demonstrated by its application with BAME staff in a UK NHS hospital trust.	
Gaines, S.O. Jr, Otermans, P., Spanoudaki, M., Aditya, D. & Chirenda, N., 2024. **Measuring relational wellbeing: Construct validity in pre-COVID-**	Study employed a series of quantitative investigations to assess the construct and criterion-related validity of	RWB is used as the central variable of interest meticulously scrutinized through quantitative methods to establish its construct validity and generalizabili	A central theoretical foundation is the concept of *inner wellbeing* - proposed by White et al. (2014). The broader discourse situated within psychology – thus defining	Two pilot studies conducted in the UK with 207 participants in the fisr pilot and 146 in the second pilot. Eligibility was being	UK, India and Greece	Social media based recruitment method (LinkedIn, Facebook, Instagram and Twitter). Online survey. Students in	Study revealed that sociocultural context was a significant predictor of relational wellbeing. Further suggests that the	Authors identified a gap in the assumption that wellbeing aspects operationalized in Global North nations could be readily

**era UK; generalizability across COVID-lockdown-era India, Greece, and UK.** *Frontiers in Psychology*, 15, Article 1342991.	an eight-item relational wellbeing scale across diverse cultural contexts. Using data from different study sites, the research applied factor analyses to evaluate the scale's dimensional structure, reliability, and cross-national applicability. Findings supported a one-dimensiona	ty as a robust, single-dimensional measure across diverse cultural contexts.	wellbeing generally as *optimal psychological functioning and experience*.	18 years and above. Main study1 in the UK=192. Main study in India=205, Greece=354, Main study2 in UK=392		the UK were also recruited through an institution-based system. Online survey data collection method. Analysed data using two statistical softwares and various construct-validity measuring tools.	theory of subjective culture, which associates an interdependent social orientation with persons in the Global East and Global South, might help explain why individuals in India and Greece generally scored higher in relational wellbeing compared to those in the Global West	generalized to Global South nations. Although the theory of subjective culture was used to interpret findings, alternative theories like the self-construal theory might be equally suited to explain sociocultural differences and similarities in relational wellbeing.

	l model of relational wellbeing revealing significant differences in wellbeing across countries.						nation of the UK.	
Nicholson, A., Hurd, F. & Ravenswood, K., 2025. ***Hauora: relational wellbeing of Māori community support workers***. *The Economic and Labour Relations Review*, pp.1–18.	The central argument of the study is that the Māori notions of wellbeing, particularly *hauora* (relational wellbeing), are fundamentally different from the Western, individualistic models	Hauora (RWB) is a holistic, relational construct that emanates from the spiritual essence of *hau* and encompasses ecological, social, and economic spheres, deeply interconnected with	The most fundamental underpinning is the Indigenous Māori Conception of Wellbeing – known as the Hauora. This concept is presented as emanating from the spiritual essence and ethic of *hau*, and traverses ecological, social, and economic	91 participants recruited. 87 interviews conducted with mostly female community workers that were 45years and above	Aotearoa, New Zealand - Maori ethnic group	Community based participatory research - used purposive snowball desgin to recruit participants and conducted indepth interviews	Māori participants highlighted culture as integral to their wellbeing because: 1. It significantly enhances their role as health care providers. 2. It helps in providing culturally approporiat	There has been a failure to fully recognize that general wellbeing and the means to achieve it are complex, multidimensional, and culturally and contextually specific. Lireature focuses on how

	and are crucial for delivering better outcomes not only for Māori but for all New Zealanders. The study demonstrates that the *hauora* of Māori Community Support Workers (CSWs) is negatively impacted by systemic factors, such as discrimination, racism, and a lack of cultural awareness and support	collective and interdependent principles. It cannot be an individual human pursuit.	spheres. The study challenges the dominance of Western, individualistic models of wellbeing in employee research, which place responsibility on individuals to manage their own wellbeing				e care. 3. It builds inner strength and resileince - preparing them for intimate care, including end-of-life support.	individual wellbeing leads to positive organizational outcomes rather than focusing on employee outcomes. It fails to address how systematic contextual factors such as gender inequity, ethnic diversity and discrimination impact employee wellbeing at both organizational and macro level.

	from employers							
Jones, D.R., Wall, T., Kenworthy, A., Hurd, F., Dyer, S., Hedges, P. & Sankaran, S., 2023. **Hiding in plain sight: Exploring the complex pathways between tactical concealment and**	This study explores the relationship between concealment and wellbeing, drawing from Scott's "hidden transcripts" and Keyes's five dimensions	The study presents relational wellbeing as a socially constructed appraisal of one's functioning in society, encompassing five key dimensions**,** which can be both enhanced	The study is grounded on the conceptualization of the "hidden transcripts" theory to explore the relationship between tactical concealment and wellbeing. It also adopts Keyes's "relationally constructed alternative" to conceptualize	Study participants were academics at different stages, ranging from 40 to 70 years of age. Disciplinary backgrounds included project management, gender	Australia, Canada, New Zealand, and UK	a collaborative ethnographic approach over a 2-year period of individual and collective inquiry.	The study reveals a dynamic, multifaceted, and often contradictory relationship between tactical concealment practices and relational wellbeing	The authors identify a paucity in critical studies that connect individual voices to concealment in diverse academic groups that have different positionalities and

**relational wellbeing.** *Organization*, 30(3), pp.473–489.	of social wellbeing through a collaborative ethnographic study within higher education, revealing a complex and often contradictory relationship between tactical concealments and relational wellbeing, presenting both generative and destructive pathways.	and complicated by the tactical use of concealment within the challenging higher education environment. They emphasize the complex, contradictory, and often hidden nature of this relationship.	wellbeing, contrasting it with an individually framed, "hedonic" view.	and diversity, creativity and arts, education, community engagement, sustainability and the natural environment, entrepreneurship, and small business financing			for academics in higher education. It offers a lens to critically explore and extend the understanding of alternative pathways to wellbeing in organizational life, emphasizing the need for critical reflexivity of concealment.	aspirations. They call for a more complex and pluralistic picture of social wellbeing beyond static models.

Pauw, L., Sun, R., Zoppolat, G., Righetti, F. & Milek, A., 2024. **May I help you? The relationship between interpersonal emotion regulation and emotional and relational wellbeing in daily life.** *Collabra: Psychology*, 10.	The study explores how individuals regulate their emotions with the help of others, a phenomenon known as interpersonal emotion regulation (IER). It specifically investigates the effectiveness of various IER strategies for improving emotional and relational wellbeing in	RWB is a central concept used to understand the quality and health of romantic relationships in daily life, particularly in the context of how individuals regulate emotions with and for each other. RWB is therefore understood as the quality of the interpersonal connection and satisfaction within a relationship. It is explicitly			Germany and Netherlands			

	daily life among romantic couples. They reviewed two preregistered ecological momentary assessment studies. Findings from the two studies illuminate the complex nature of interpersonal emotion dynamics and how enacted versus perceived regulatory behaviors can lead to	linked to feelings of closeness and relationship satisfaction.						

	different outcomes							
Katisi, M., Tonheim, M., McGregor, S. & Mubeen, F., 2024. **Narratives of symbolic objects: Exploring relational wellbeing of young refugees living in Scotland, Finland, and Norway.** *Social Sciences*, 13(1), p.43.	Through using objects as representations of relational wellbeing, this paper generates narratives of young refugees and members of their social networks to generate relational wellbeing.	Relational wellbeing described as constituted by three aspects - feeling good, being connected and having enough, which the paper viewed as inseperable. Authors indicate this as a challenge for researchers	The paper utilises White's (2015) conceptualisation of relational wellbeing. However still in the utilisation of the construct approaches it as an outcome variable and less so about an orientation to understanding wellbeing. In many instances referring to resulting in	Now 18 years and above, but arrived as unaccompanied minors.	Young refugees settled in Scotland, Finland and Norway. From Sri Lanka, Somalia, Afghanistan, Eritrea, Uganda, Iran, Republic of Congo, DRC, Sudan,	Longitudinal qualitative research using objects, art and ecomaps to map a timescape of relational wellbeing for young refugees	Identified three overlaps revealing complexities and similarities in how wellbeing is experienced by yougn refugees: overlaps in time and space; overlaps between old and new social ties; and	Practitioners should be alert to the interconnectedness of past and present experiences as key to wellbeing. Not a lot of work using this approach as well as utilising objects to convey and as vessels of meanings

	Using these objectis revealed hoew relational wellbeing was experienced and expressed by these individuals. In finding overlaps in experiences and expressions, captured by the three themes of fiscussion: overlaps between old and new social ties; time and spce; and three constructs of	adn practitioners to develop complex research and intervention methods that can capture these tapestries of young refugees' experiences of relational wellbeing. Approach relational wellbeing mostly as an outcome variable.	relational wellbeing.		Ethiopia, Pakistan, Syria and Myanmar		overlaps between the three constructs of relational wellbeing approach of being connected, having enough and feeling good. Objects are useful tools to elicit complex emotions and interpretation around what contributes to youth wellbeing. Relational wellbeing transcends and	which in this study provided agency to tell stories that would otherwise not be told. The significance of interacting with old and new social ties shows the need for these ways of relating to be maximised by policy makers and practitioners to enhance young people's relatinoal wellbeing in host countries.

	relational wellbeing, the paper concludes that these overlaps have implications for a relational wellbeing approach in theory and practice.						overlaps through time and space and relationships and their meanings are not static and can reflect multiple belongings. They found that although all dimensions were present, the material dimension of having enough to largely be lacking in the participants narratives abour their objects.	

Haswell, N., 2023. **Nature and belonging in the lives of young refugees: A relational wellbeing perspective.** *Social Sciences*, 12(11), p.611.	Explores the relationship between nature contact, wellbeing and belonging in the resettlement experiences of young refugees in Finland. Using a relational wellbeing approach, considers how subjective, material and relational dimensions of wellbeing arise and	Conceptualises belonging as follows: people can have and develop a sense of belonging to particular nature places, as well as to people, communities and cultures within and through encounters with nature places. People's sense of belonging emerges from the holistic, simultaneously experienced, physical,	Refers to White and Jha and indicates that it draws from their conceptual appraoch to wellbeing that draws attention to its material, subjective and relational dimensions as well as the interconnections between them.	Male and female 18-30 year old former unaccompanied asylum seeking minors in receiving countries of Finland, Norway and Scotland (UK).	Young refugees settled in Finland.	Indicates that it uses a relational wellbeing approach to implement a qualitative invesitgation that makes use of participant-made artworks and semi-structured interviews to illuminate the different ways in which refugees encounter nature in their past, preent and	Nature encounters can foster a sense of belonging in three ways: through restoration and attachment in the present, through maintaining links with the past, and through shaping desires about a future in which to thrive. These three aspects represent particular interrelatio	Still reflects on relational wellbeing as something that is generated and an outcome, as evidenced in reference to "considering refugees' sense of belonging in Finland as part of the relational wellbeing generated, in part, from their encounters with nature, these three aspects of belonging represent particular interrelations between

	interrelate within refugess encounters with nature and how these encounters link with refugees developing sense of belonging to people and places in Finland.	affective, cognitive and social dimensions of encoutering people, objects and places. The strength of people's sense of belonging or not depends on the extent to which those dimensions generate circumstanc es of feeling good, having enough and being connected. Sees belonging as not external to but an aspect of a				imagined future.	ns between subjective, material and relational dimensions of refugees/ wellbeing. The subjective, seen in the way that the encounters made refugees feel good through positive feelings and thoughts generated by and within the encounter. The material, seen in their having	subjective, material and relational dimensions of refugees' wellbeing". This paper focuses on the less studied links between the wellbeing refugees derive from nature contact and their sense of belonging to their new physical, social and cultural environment s. A focus primarily on the positive aspects of relational wellbeing, meant that

		person's relational wellbeing.					enough access to, as well as enough time and resources to visit particular nature places with characteristics important to them. The relational, seen in the way that encounters made them feel connected, either with other people through social interactions in nature or with	potentially significant negative aspectw of the nature-wellbeing nexus remained unexplored.

							nature itself.	
Molla, T., 2023. **Racial othering and relational wellbeing: African refugee youth in Australia.** *Social Sciences*, 12(11), p.609.	Paper demonstrates the pervasive culture of racial othering through media identifications of African youth with criminality and gang	Concentrates on tqwo aspects of relational wellbeing: the capacity to move in public without fear or shame and the ability to feel a sense of belonging to the place	Draws from capability approach and relational perspective with interdisciplinary literature on racial othering. However, the capability approach is seen as providing only a partial view of the interplay between social	Youth from African heritage settled in Australia	Australia	QUalitiatve data through semi-structured interviews with 44 African-heritage young people who arrived in Australia on a permanent	Findings showed that the racialisation of youth violence undermined important aspects of African youth's wellbeing: dignity (freedom to interact	In refugee resettlement and integration policies and programs substantive opportunities for newcomers to relate with others and be recognised for who they

	violence and illustrates impacts on young people's wellbeing through data from interviews with Africa refugee youth.	where one lives in. Tendency to deal with relational wellbeing as outcome indicator illustrated in the sentence "how has the racialisation of violence affcted the opportunity for African refugee youth to achieve relational wellbeing".	arrangements and wellbeing, whereas the relationa dimnesions of wellbeing emphasises the importance of social context and how this promotes or undermines people's scope to experience wellbeing.			humanitarian visa from eight main countries: Congo, Eritrea, Ethiopia, South Sudan, Ghana, Liberia, Somalia and Sierra Leone. Covering educational opportunities and experiences as well as their sense of safety and belonging.	with others without fear) and belonging (opportunities to participate in their communities). Young people have diminised wellbeing opportunities when they lack equal worth and respect and face difficulties in establishing meaningful relationships with others - when they are not free	are is required. Shows the importance of adding a relational approach to understanding wellbeing of young refugees.

							from alienation, deprivation and domination .	
Seda, F.S.S.E., Kurniawan, K.N. & Pera, Y.H.T., 2023. **Social inclusion challenges and the future of relational wellbeing: The case of Indonesia and South-Korea.** *Social Indicators*	Intends to fill gap in examining how vertical and horizontal forms of social inclusion have impacted on wellbeing in its relational forms: the capacity to	Quotes White in asserting that the research aims to bring a different perspective on individual happiness that is specifically viewed through a relational lens. Relational wellbeing is	Aims to test the Easterlin paradox that find that as a society becomes more economically developed, citizens' wellbeing decreases. However, relational wellbeing is understood and investigated as a dependent variable, with	Household heads (18-64 years old)	Indonesia and South Korea	Quantitative comparative study of INdonesian and South Korean society.Univariate correlation between horizontal and verticxal dimensions of social inclusion in	A sustainable and blanaced form of relational well-being does not only consist in economic attributes vertically, but also social-horizontal dimension,	Inclusive government policies at the personal, relational and societal level is very fundamental to create sustainable wellbeing . Tendency to look at relational wellbeing as dimension of

Research, 165, pp.309–332.	trust others, the degree of interaction and pro-activity in communal participation.	defined as the individual's sense of social happiness which is cultivated through subjective perceptions of trust, personal interactions with fellow individuals and social engagement in communal activities. Three indicators defined: trust or perceptions, interactions and participation.	vertical (assets, income level, occupational background and educational level) and horizontal (gender group, religious affiliation, region of origin, race/ethnicity/nationality) dimensions of social inclusion are independent variablea			the context of relational wellbeing.	which is manifested through social capital and cohesion facilitated by cultural, religious and gender groups in local communities. Economic (material) factors are more dominant in explanining relational wellbeing at individual level, while social (non-material) factors are dominant	overall wellbeing.

							as explanations at the community level.	
